# The shadow of the future promotes cooperation in a repeated prisoner’s dilemma for children

**DOI:** 10.1038/srep14559

**Published:** 2015-09-29

**Authors:** Peter R. Blake, David G. Rand, Dustin Tingley, Felix Warneken

**Affiliations:** 1 Boston University, Department of Psychological and Brain Sciences, 64 Cummington Mall, Boston, MA 02215; 2 Yale University, Department of Psychology, Department of Economics, School of Management, 2 Hillhouse Ave, New Haven, CT 06520; 3 Harvard University, Department of Government, 1737 Cambridge St, Cambridge, MA 02138; 4 Harvard University, Department of Psychology, 33 Kirkland Street, Cambridge, MA 02138.

## Abstract

Cooperation among genetically unrelated individuals can be supported by direct reciprocity. Theoretical models and experiments with adults show that the possibility of future interactions with the same partner can promote cooperation via conditionally cooperative strategies such as tit-for-tat (TFT). Here, we introduce a novel implementation of the repeated Prisoner’s Dilemma (PD) designed for children to examine whether repeated interactions can successfully promote cooperation in 10 and 11 year olds. We find that children cooperate substantially more in repeated PDs than in one-shot PDs. We also find that girls cooperate more than boys, and that children with more conduct problems cooperate less. Finally, we find that children use conditional cooperation strategies but that these strategies vary by gender and conduct problem rating. Specifically, girls and children with few conduct problems appear to follow an altruistic version of win-stay, lose-shift (WSLS), attempting to re-establish cooperation after they had defected. Boys and children with more conduct problems appear to follow a Grim strategy, defecting for the duration after the partner defects. Thus we provide evidence that children utilize the power of direct reciprocity to promote cooperation in strategic interactions and that, by late elementary school, distinct strategies of conditional cooperation have emerged.

Cooperation is a central feature of human societies but is a challenge because it requires individuals to pay costs to benefit others. Direct reciprocity is one mechanism that can support cooperation between genetically unrelated individuals: when people interact repeatedly, it can be worth paying the cost of cooperation today in order to gain reciprocal cooperation from one’s partner tomorrow^1^,^2^,^3^. The role of direct reciprocity has been studied extensively in adults using the repeated prisoner’s dilemma (PD), a standard paradigm for exploring human cooperation^4^. In a typical PD, pairs of individuals simultaneously decide whether to cooperate or defect and payoffs are determined by their combined decisions. When both parties cooperate, they maximize their combined payoff. But each individual has an incentive to defect and achieve a higher individual payoff, leaving their cooperating partner with the lowest payoff. When partners will only interact once, the temptation to defect makes defection the dominant strategy for both parties and results in a lower combined payoff.

Direct reciprocity provides one solution to the problem of defection in the PD. In one-shot PDs, pairs interact only once and reciprocity is not possible. However, when pairs engage in repeated interactions, reciprocal strategies can lead to increased cooperation: if your partner’s cooperation in the future rounds is contingent on you cooperating today, even selfish people may cooperate (given a large enough probability of future interaction)^5^,^6^. Experimental comparisons of anonymous adults playing PDs with different time horizons have shown that, for the same payoff matrix, people cooperate more in games with a higher potential for future interactions with the same partner^4^,^7^. Analyses of strategies used in repeated PDs show that most adults either use a conditional strategy of tit-for-tat (TFT) or simply always defect (ALLD)^8^. The “shadow of the future” thus can promote cooperation via conditional play, even when players remain anonymous to each other.

While the repeated PD has been used extensively in experiments with adults^7^,^8^,^9^,^10^,^11^,^12^,^13^, and children’s play in other economic games has been explored in recent years^14^,^15^,^16^,^17^,^18^,^19^,^20^,^21^,^22^,^23^,^24^, repeated PD experiments with children remain rare. Most importantly, direct comparisons of children’s play in PDs of different lengths are lacking. (The few prior studies which have examined play in repeated PDs have not compared play to games of different lengths^25^, and have either been non-anonymous^26^,^27^ or involved more than 2 players^28^; in the latter case, cooperation is not expected to succeed even in the presence of repeated interactions). In the current experiment, we compared children’s play in a fixed-length repeated PD to play in a series of one-shot PDs. In the repeated PD, children were paired with an anonymous partner for six rounds, and in the one-shot PD, children were randomly re-matched with a new anonymous partner after each round and the history of the partner’s prior decisions were not given. The key difference between these two conditions is that direct reciprocity is possible in the repeated game but not in the one-shot games. Thus, comparing play in one-shot versus repeated PDs can reveal whether children recognize the potential for direct reciprocity in repeated games and whether they use strategies of conditional cooperation to encourage future cooperation.

One barrier to the use of repeated PDs with children is the difficulty of explaining and presenting the decision problem they will face. To make the PD payoff structure more intuitive, we designed a novel, graphical interface to present the prisoner’s dilemma (Fig. 1). We tested pre-adolescent children (mean age 11.6 years; N = 64; 44 females) in the 5^th^ and 6^th^ grade of an elementary school in the US (the gender imbalance reflected the male/female ratios in the classes). Classrooms were assigned to play a series of either one-shot or repeated PDs (1,790 total decisions). In order to isolate the role of direct reciprocity in children’s strategic decisions, we maintained anonymity between partners and compared one-shot and repeated PDs.

We were also interested in whether children’s individual characteristics or traits influenced their decisions in the PD. For example, in some PDs girls cooperate more than boys^29^ but other studies have found no differences^28^. Recent research has also demonstrated that children with behavioral problems approach strategic games differently^30^,^31^. Problems such as conduct disorder typically appear in late elementary school and can persist into adulthood^32^. To explore the possibility that behavioral issues might influence children’s decisions, we asked parents to complete the Strengths and Difficulties Questionnaire (SDQ)^33^,^34^, a measure widely used to assess children’s behavioral problems on five dimensions: Prosocial Behavior, Conduct Problems, Emotional Problems, Hyperactivity and Peer Problems^35^ (see Table S1 for full questionnaire).

## Results

We first examined the overall effect of direct reciprocity by comparing the probability of cooperation in the one shot and repeated games. We used logistic regression models with clustered standard errors at the level of the individual and the pair (Stata v.13.1). No grade differences were found and thus both grades were combined for analysis. Children cooperated more in the repeated compared to the one-shot PD (coeff = 0.563, p = 0.013), an effect that also held when examining only the first round of play for each type of game (coeff = 1.216, p = 0.023) (Fig. 2). Regressions including trial number found that cooperation declined over the course of the session (coeff = −0.041, p < 0.001) in both repeated and one-shot games (no interaction between decision number and repeated-game dummy: coeff = 0.011, p = 0.406), but that cooperation remained higher in the repeated games (coeff = 0.675, p = 0.003) when controlling for trial number (Figure S1). These results demonstrate a “shadow of the future” effect in school-aged children. Children recognized the strategic difference between one shot and repeated PDs, even in the first round of play, and responded to the incentives created by direct reciprocity in the repeated game.

We next ran a regression adding gender and the five SDQ variables (and including a Bonferonni correction for 6 multiple comparisons). Results showed that boys cooperated less than girls (coeff = −0.941, p = 0.002 corrected), and that children rated higher on Conduct Problems tended to cooperate less (coeff = −0.278, p = 0.012 corrected) (Fig. 3); all other SDQ measures were non-significant (p > 0.10 corrected). A regression including interactions between a repeated-game dummy and each individual-difference measure found no significant interactions (all ps > 0.20), indicating that the gender and Conduct Problem effects applied to both one-shot and repeated games. To interpret these coefficients, we calculated the odds ratios for gender and conduct (see supplement for details). Girls were almost 2.5 times more likely to cooperate than boys and a one point increase in conduct problems score predicted a 15% decrease in cooperation.

Finally, we performed a more detailed analysis of children’s strategies in the repeated games. Given that children were aware that each repeated game lasted six rounds, they may have engaged in a process of backwards induction^36^,^37^, switching from cooperation to defection as the end of each repeated game approached. We did not find evidence of this strategy: cooperation was not significantly lower in the last round compared to the second-to-last round (coeff = −0.110, p = 0.362), nor was there a significant decrease in cooperation over rounds when considering rounds 2–6 within each game (coeff = −0.008, p = 0.868). Thus, although cooperation in the repeated games classrooms decreased over the course of the entire testing session, children did not appear to decrease their cooperation in the later rounds of each separate game. Although end-game effects have been found for adults playing repeated games, children may not have engaged in the more sophisticated form of strategic thinking required for backwards induction^38^.

Instead, we found evidence that children were using conditional strategies (Fig. 4). They were significantly more likely to cooperate when their partner cooperated in the previous round (44.9% C) than when their partner defected in the previous round (27.4% C) (coeff = 0.772, p < 0.001). An interaction between the player’s own previous move and the partner’s previous move was significant (coeff = 2.033, p < 0.009) and offers further insights into the strategies children used. When children had cooperated in the previous round, cooperation was contingent on the partner’s previous move (52.8% C in response to partner’s C, 11.1% C in response to partner’s D; coeff = 2.192, p = 0.001). Thus, mutual cooperation in the prior round encouraged continued cooperation, whereas being exploited (child C, partner D) in the prior round evoked defection. When children themselves had defected in the previous round, the partner’s decision did not produce this effect; rather, children showed an intermediate level of cooperation (38.9% C in response to partner’s C, 35.2% C in response to partner’s D; coeff = 0.158, p = 0.427). Interestingly, this pattern means that after mutual defection children were more likely to return to cooperation (35.2%) than when they had been exploited in the prior round (11.1%). In sum, when direct reciprocity was possible, children relied on conditional strategies to a substantial degree.

Conditional cooperation also varied by individual characteristics of the children. One factor was the child’s gender (Fig. 5a). Both girls and boys responded to mutual cooperation with cooperation, but tended to defect after they had cooperated and the partner defected (i.e. after they had been exploited; child C, partner D). However, differences emerged when the children themselves had defected in the prior round. After mutual defection, girls were more likely to cooperate than boys (girls, 44.5% C; boys, 24.6% C; coeff = −0.901, p = 0.019) . In addition, after the child had defected and the partner cooperated (child D, partner C), earning the child the highest payout, girls were again more likely to cooperate than boys (girls, 46.3%; boys, 24.1%; coeff = −1.000, p = 0.028). Thus, although girls tended to cooperate more than boys overall, this difference was driven mostly by cooperation after the actor’s own defection.

A child’s level of conduct problems was also important for conditional strategies (Fig. 5b). We created conduct problems subgroups by separating children with scores in the “abnormal” range of the scale into a High Conduct Problems group (*N* = 3) and children with lower (typical) scores into a Low Conduct Problems group (*N* = 27). The proportion of children in the High Conduct Problems group (10%) matches that expected for a community sample^34^, however, all of the children in this group in the current study were girls. The results comparing the two conduct problems groups mirrored the pattern seen for girls and boys. Children in both the Low and High conduct problems groups responded to mutual cooperation with cooperation and defected after they had cooperated and the partner defected (child C, partner D). Differences emerged between these groups when the children themselves had defected in the prior round. Children with Low conduct problems were more likely to cooperate than children with High conduct problems after mutual defection, although this difference was not significant (33.4%, Low; 23.1% High; coeff = −0.516, p = 0.263). However, when children had defected and the partner had cooperated in the prior round (child D, partner C), children with Low conduct problems were marginally more likely to cooperate (38.7%, Low; 15%, High; coeff = −1.275, p = 0.051). Thus, children who scored in the abnormal range for conduct problems (High) were less likely to try to re-establish cooperation after they themselves had defected.

Interestingly, the decisions made in the repeated game by boys and by children with high conduct problems resembled a strategy known as Grim, which is less forgiving than TFT: Grim cooperates until the partner defects, and then defects forever^4^. Therefore, unlike TFT, Grim does not cooperate after child D, partner C. Girls and children with low conduct problems, on the other hand, employed a strategy that resembled a combination of TFT and Win-Stay, Lose-Shift (WSLS)^39^. WSLS performs well in repeated games where errors are possible^39^, but is rarely found in adult versions of the PD^4^,^8^,^11^. When using WSLS, players stay with their previous decision when it has produced a high payoff for themselves. Thus actors continue to cooperate after mutual cooperation (CC) and continue to defect after they defect and the partner cooperates (DC). In contrast, after losing decisions, actors shift to the opposite move: they switch to defection after they have been exploited (CD) and switch to cooperation after mutual defection (DD). Girls and children with low conduct problems appeared to follow WSLS except in the case of DC, when they received the highest payoff. After DC, these children returned to cooperation to a high degree, perhaps reflecting a more altruistic version of win-stay, lose-shift. In sum, children’s individual characteristics proved an important factor in determining the conditional strategies they used in the repeated PD.

## Discussion

This study provides the first evidence that direct reciprocity promotes cooperation among children engaged in a repeated prisoner’s dilemma, leading to more cooperation when future interactions are possible compared to when they are not. Increased cooperation in the repeated relative to the one-shot game was apparent even in the first round of play in each condition. Children thus recognized that the fact of future interactions with the same partner presented greater opportunities for cooperation, the so-called shadow of the future. Importantly, children remained anonymous to each other during the experiment, allowing us to isolate the role of direct reciprocity in strategic decisions. This stands in contrast to prior games in which children could identify each other, raising the possibility that concerns about reputation outside of the game context and personal history with the partner had influenced decisions.

This study also demonstrates that children as young as 10 years of age engage in conditional strategies of cooperation in the repeated PD. Prior experiments have found little evidence of conditional cooperation in PDs with children. For example, in a multiple-player social dilemma (Public Goods Game), 10 to 16 year olds did not respond reciprocally to their partner’s decision; most notably, cooperation did not increase after mutual cooperation^28^. The current study adds to these results by demonstrating that in an anonymous, dyadic, repeated PD children *do* use conditional strategies, cooperating most after mutual cooperation and least after they cooperated and the partner defected in the prior round, leading to the lowest payoff to the child. This may be due in part to the fact that direct reciprocity can successfully maintain cooperation in dyadic relationships, whereas in multiplayer games, even among adults, cooperation tends to decrease over time^40^.

Importantly, the conditional strategies used by children varied based on two individual characteristics: gender and conduct problems. Girls were generally more cooperative than boys and were more likely to cooperate after they had defected in the prior round. What factors underlie this difference in behavior remains an open question. To some extent, children may have been responding to either the expected or actual behavior of partners of the same gender. In most of the repeated games girls played girls and boys played boys, and the children knew this before starting the game. However, there was one repeated game in which the partner could be either the same or opposite gender, and children used the same strategies as in the single gender games. This suggests that the strategies used by girls and boys may be stable across different kinds of partners and that girls may be genuinely more forgiving of defection. However, caution is warranted in interpreting this result due to the small sample size and the relatively small number of boys in the sample. In addition, gender differences in children’s prosocial behavior vary across studies and tasks and often fail to replicate^41^. Thus, replication studies are needed to ensure that the gender differences in strategies are stable and, if so, to understand what cognitive and social factors drive the differences.

Intriguing differences also appeared for children scoring high on the conduct problems scale. Children who were rated by their parents as having more conduct problems tended to cooperate less in both the one-shot and repeated PD. For the repeated games, this subset of children was more likely to defect overall and used less forgiving conditional strategies that resembled Grim. The finding that children with different degrees of conduct problems use different strategies in the repeated PD is striking for two reasons. First, it shows that distinct forms of conditional cooperation have emerged by late elementary school for children with different behavioral profiles. Research with adults has found some evidence of links between personality traits and cooperation in the PD^42^,^43^,^44^, but to our knowledge no studies have examined children’s traits or behavioral profiles in relation to their decisions in the PD. Our results show that individual differences in cooperative strategies emerge much earlier than adulthood.

Second, the connection between children’s strategies in the repeated PD and their behavior outside of the experimental context suggest that this task can be an important tool for studying the development of behavioral problems. Around 10 years of age, behavioral problems increase dramatically and often persist into adulthood^32^. Different sub-types of conduct problems are evident before age 10 and problems manifest differently for boys and girls^45^. The current study thus highlights the potential for using intuitive versions of the repeated PD to better understand how problematic behavior emerges in complex social situations and what factors encourage cooperation among children with different behavioral profiles.

Although we cannot determine the exact motivations that underlie the different strategies used by children with conduct problems, prior studies offer some insight. For example, one study used a one-shot Trust Game with a group of children clinically diagnosed with conduct disorder^31^. In this task, an investor must decide how many points to pass to a trustee; the points are tripled (in this case) and then the trustee decides how many points to send back to the investor. Children with conduct problems in the investor role tended to send less to the trustee compared to children without problems, demonstrating low trust. In addition, in the trustee role, the clinical group sent less back to the investor compared to the control group, demonstrating low trustworthiness. When asked to explain their decisions, children with conduct problems often referred to mistrust of the partner or their perception that the partner’s decision was hostile. Thus, in the current study, the low rates of cooperation and forgiveness among children with conduct problems may have been due to a general mistrust of their partners.

In summary, the current study makes two key contributions. First, we demonstrate that children as young as 10 years of age take advantage of the potential for direct reciprocity to enhance cooperation in the prisoner’s dilemma. When repeated interactions are possible, children engage in conditional cooperation, even though the interactions are anonymous. Thus, by late elementary school children already possess the cognitive capacities necessary for strategic cooperation in a complex social dilemma. Second, we find that the particular strategies used by children vary based on their gender and by their behavior in the world outside of the experiment. This latter finding has potentially important policy implications. If teachers are interested in promoting cooperation within the classroom, different approaches may be needed for girls and boys and for children with behavioral problems in particular. In addition, given that children with conduct problems show distinct patterns of play in the repeated PD, this task may prove an important tool for future study of the psychological mechanisms that lead to lower cooperation in this group.

## Materials and Methods

We tested 5^th^ and 6^th^ graders recruited from a US elementary school. The school site was selected because it is a science and technology charter school in which children gain experience using computers as early as the first grade. The school had fleets of laptop computers that could be rolled into each classroom and a wireless network that could support the online game. These features allowed us to test children within their school.

The overall mean age was 11.6 years and the age range was 9.75–13.33 years. One sixth grader missing age data was assigned the mean age for the grade. The school’s mission is to serve minority children and while only 60% of parents provided demographic information, 80% of those responding described themselves as Black or African-American. Roughly half of the parents had completed some college and the median household income was $42,000. Parental consent was obtained in writing and child assent was obtained the day of the experiment. All consent and experimental procedures were approved by the Institutional Review Board at Harvard University (IRB #F20073). The methods were carried out in accordance with the approved guidelines.

**Figure 1:**
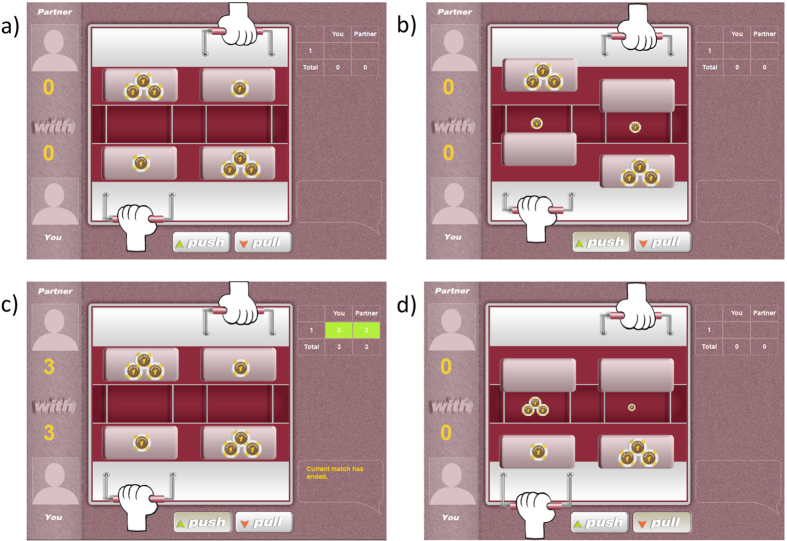
Prisoner’s Dilemma interface. Two players face each other on screen, actor on bottom, and can either push or pull their tray by clicking buttons for these options. Pushing the tray delivers three coins to the partner and causes one coin to fall into an abyss. Pulling the tray delivers one coin to the actor and causes the three far coins to fall into the abyss. Panels a-c show the sequence of play from the (**a**) starting position, (**b**) the result of both players choosing push (CC), and (**c**) the payoffs to both players shown in the right side history bar. Panel (**d**) shows the result of the actor pulling and the partner pushing (DC).

**Figure 2:**
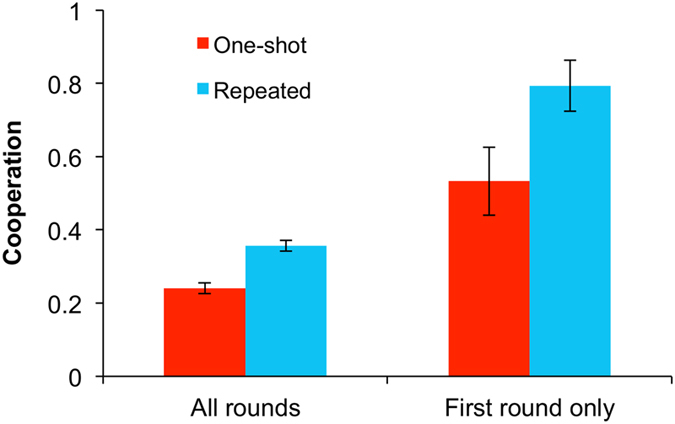
Direct reciprocity promotes cooperation in school-aged children. Shown is the frequency of cooperation in all rounds of the 1-shot versus repeated games and in the first round of play only. Error bars indicate standard errors of the mean.

**Figure 3:**
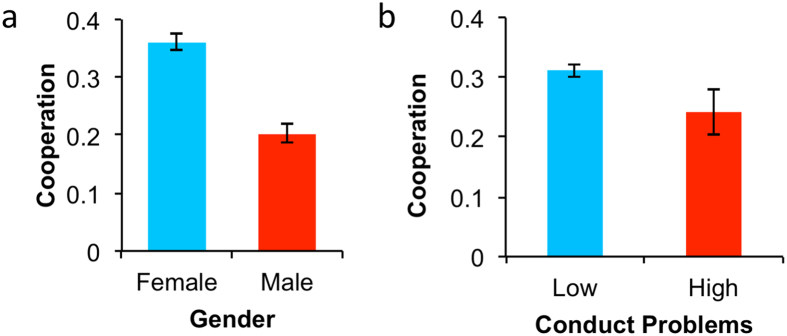
Boys and those with conduct problems cooperate less in the PD. Shown is the frequency of cooperation across all games for (**a**) girl and boys and (**b**) by Conduct Problems, with High representing children rated in the abnormal range of the scale and Low representing children in the typical range of the scale. Error bars indicate standard errors of the mean.

**Figure 4:**
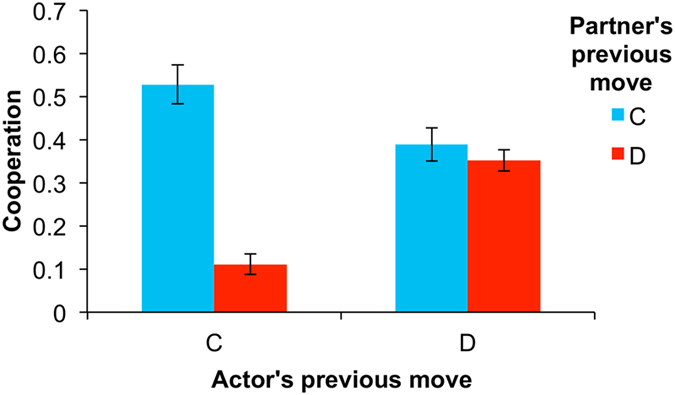
Children use conditional cooperation strategies in repeated games. Shown is the frequency of cooperation in repeated games as a function of the partner’s move in the previous round and the actor’s own move in the previous round. Error bars indicate standard errors of the mean.

**Figure 5:**
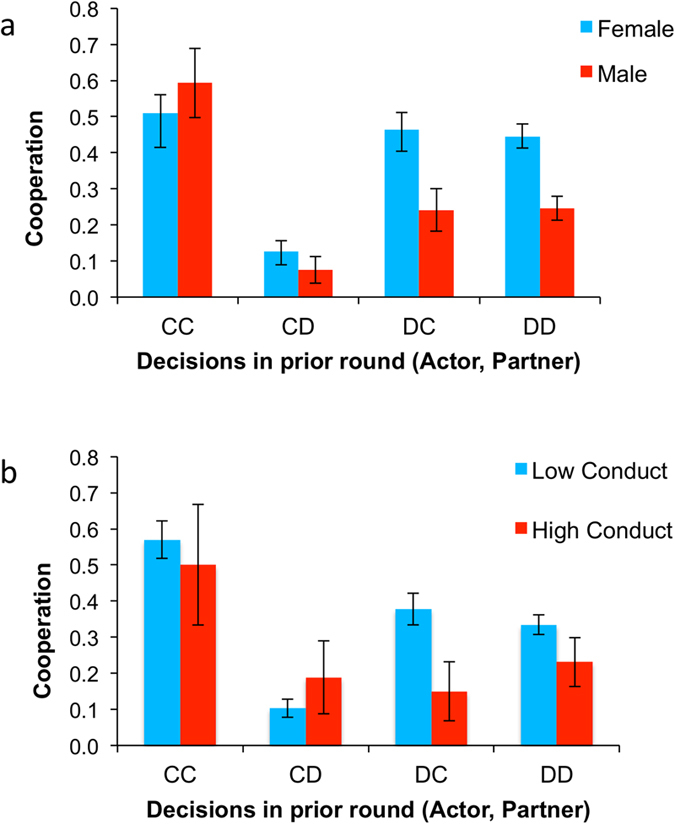
Different conditional strategies by sub-group. Shown is the frequency of cooperation in repeated games as a function of the actor’s and the partner’s decision in the previous round. Strategies varied by gender (**a**) and conduct problem group (**b**). Error bars indicate standard errors of the mean.

Experiments were conducted using a standard computerized version of the PD (www.econvision.com) with a novel interface for children (Fig. 1). In the one-shot version of the game, children played a series of one shot games (mean # trials = 26) and in the repeated version they played a series of 6-round, fixed length games (mean # trials = 30; median # games = 5). In all classrooms, we ran as many games as time would allow which lead to the difference in the mean number of trials between the one shot and the repeated games. To address concerns that this difference can explain the differences between the one shot and repeated games, we re-ran all of the analyses comparing the two games using only the first 24 trials of each game. This subset analysis did not materially change the results.

Three 5^th^ grade and two 6^th^ grade classrooms were tested. The first classroom tested was randomly assigned, by a coin flip, to receive either the repeated PD or the one-shot PD. The next classroom for each grade played the other game; the third 5^th^ grade class received another repeated game. Within each classroom, boys and girls played separate games: boys played with boys and girls played with girls, and the children were told that this was the case. In the third 5^th^ grade repeated game, we conducted a mixed gender game due to a smaller number of participants. Adding this factor to our regressions did not produce different results. (See Table 1 for final sample size by gender and game). Children used laptop computers while sitting at their regular desks with cardboard barriers added to create cubicles to reduce interactions outside of the games; partners remained anonymous in all games.

The interface showed two hands, one for each player, that could either push or pull a tray (Fig. 1). Clicking a Push button delivered three coins to the partner (Cooperate) and clicking the Pull button delivered one coin to themselves (Defect). This created a payoff matrix of DD = 1, CD = 0, DC = 4, and CC = 3 (Table 2). Decisions were made simultaneously, and the trays moved after both children had made their decision. Payoffs to each player for each round were visible on a side bar. To ensure comprehension, a cartoon video described the characteristics of the game, comprehension checks were performed and practice trials were conducted prior to the start of the experiment (for full procedure see Supporting Information).

To incentivize children, we told them that the total points they earned during the study could be used to “purchase” prizes from the experimenters the following week. Children were shown several prizes that would be available, and it was emphasized that with more points they could acquire more and better prizes. The prize purchase day occurred about one week after the testing session for each classroom.

To measure children’s behavioral problems we used a standard parent report questionnaire, the Strengths and Difficulties Questionnaire (SDQ; Goodman, 1994, 1997) (see Table S1 for full questionnaire). The SDQ consists of 25 questions that assess five constructs of child behavior: Prosociality, Conduct Problems, Emotional Problems, Hyperactivity and Peer Problems. This instrument is widely used to screen for childhood psychopathology. The SDQ was sent home to parents along with the consent forms. Fifty-eight parents completed the SDQ (91%).

## Additional Information

**How to cite this article**: Blake, P. R. *et al.* The shadow of the future promotes cooperation in a repeated prisoner's dilemma for children. *Sci. Rep.* **5**, 14559; doi: 10.1038/srep14559 (2015).

## Supplementary Material

Supporting Information

## Figures and Tables

**Table 1:** Sample size for repeated and one-shot games by gender.

	Repeated	One Shot	Total
Female	22	22	44
Male	12	8	20

**Table 2:** Prisoner’s Dilemma Payoffs.

		Player B	
		C	D
Player A	C	3,3	0,4
	D	4,0	1,1
