# Supplementary material for: The shadow of the future promotes cooperation in a repeated prisoner’s dilemma for children
Source: Sci Rep. 2015 Sep 29;5:14559. doi: 10.1038/srep14559 (PMC4586758; doi:10.1038/srep14559)

Supporting Information for

The shadow of the future promotes cooperation in a repeated prisoner's dilemma for children

Peter R. Blake[a], David G. Rand[b], Dustin Tingley[c] and Felix Warneken[d]

[a]Boston University
Department of Psychological and Brain Sciences
64 Cummington Mall
Boston, MA 02215

[b]Yale University
Department of Psychology, Department of Economics, School of Management
2 Hillhouse Ave
New Haven, CT 06520

[c]Harvard University
Department of Government
1737 Cambridge St
Cambridge, MA 02138

[d]Harvard University
Department of Psychology
33 Kirkland Street
Cambridge, MA 02138

Additional Methods

Pilot testing

The interface we used for the PD was new and untested; therefore, we conducted pilot tests with three 4th grade classrooms. The initial repeated game design had a probability of continuation of 1/6, represented by a die roll on screen after each round. The children were confused by this aspect of the game and teachers confirmed that the children at this age do not comprehend probabilities. Therefore, we modified the repeated game to be fixed length. We pilot tested this version of the repeated game and the one-shot games with different 4th grade classrooms and tested the cartoon video used to introduce the game.

Procedure

Children were first asked if they would like to play the game (an approved assent procedure in addition to parent consent). Two children opted not to participate. Children then watched a short video introducing the interface and describing the basic features of either the one-shot or the repeated game, depending on condition: i) children would not know who their partner in the game was; ii) they would play a series of games with a different partner each time; iii) boys played with boys and girls played with girls (where applicable); iv) players would earn points during the game that they could use to "purchase" prizes on a different day. In the video, children in the one-shot condition were told that they would play multiple, single interaction games, changing partners for each game. Children in the repeated condition were told that they would play several 6-round games, playing with the same partner for each game and then changing to a new

partner for each new game. After the video, children were asked a series of comprehension questions to ensure that they understood the different payoffs from each combination of decisions. These questions were based on questions used in adult versions of the game and were as follows:

> If you click push and the other person clicks push how many do you get? And how many does the other person get?
>
> If you click push and other person clicks pull how many do you get? And how many does the other person get?
>
> If you click pull and the other person clicks push how many do you get? And how many does the other person get?
>
> If you click pull and other person clicks pull how many do you get? And how many does the other person get?
>
> Which row has the most total points if you add the whole row together? (referring to the history of play bar from the demonstration video)
>
> How many rounds do you play with the other person?
>
> For each game do you play with a new person?

Next, children did practice trials with the live interface to ensure that they understood the consequences of each combination of decisions. Once children had logged into the system, the experimenter instructed all of the children to click the push or pull button (order counterbalanced for the repeated and one-shot classes); then all to click the other button. Next, the experimenter instructed half the class to click push, and half to click pull; then the opposite; and last two again dividing the class differently, after

research assistants checked each child's payoffs, to ensure that all had experienced all four possible payoffs.

Children were then shown the prizes that could be earned in order to incentivize them to take their decisions seriously. They were allowed to ask questions before the testing began and were told that talking was not allowed once the study started. The experimenters returned about one week after the testing session to allow children to select prizes based on the number of points earned – the "prices" of the prizes were scaled so that all children received at least one prize.

Before starting the testing session, barriers were set up on tables to reduce interaction between the children and prevent them from seeing each other's screens. Children were reminded that there was no talking allowed once the study began. The experimenter then announced that the game had started and that children should decide what to do.

When there were an odd number of boys or girls in a class, one research assistant played a TFT strategy against the odd one out. Children were not told this and the RA decisions are excluded from the analyses.

The experiments were conducted over a four week period during regular classroom sessions. The children played as many games as possible within the 45 minute window of the class time. The final sample included 64 children (44 girls) with 34 playing repeated games and 30 playing one shot games (Table 2). 1,790 decisions were analyzed.

Strengths and Difficulties Questionnaire

The SDQ was sent home to parents along with the consent forms. Fifty-eight parents completed the SDQ (91%). The children in our game were from a typical classroom sample, and we did not have clinical diagnoses for the children. However, the SDQ scoring site (http://www.sdqinfo.org/) notes that for community samples, 10% of the children typically fall in the abnormal range of the scale. This was the case for children in our sample on the Conduct Problems scale, and these children constitute the High Conduct Problems group. See Table S1 for the full questionnaire with the Conduct Problems items in bold.

Table S1. Strengths and Difficulties Questionnaire (Goodman, 1994, 1997). The questionnaire consists of 25 questions to which parents can respond either Not True, Somewhat True or Certainly True. Items on the Conduct Problems scale are highlighted in bold; (R) means the item is reverse coded.

1. Considerate of other people's feelings
2. Restless, overactive, cannot stay still for long
3. Often complains of headaches, stomach-aches or sickness
4. Shares readily with other children, for example toys, treats, pencils
5. **Often loses temper**
6. Rather solitary, prefers to play alone
7. **Generally well behaved, usually does what adults request (R)**
8. Many worries or often seems worried
9. Helpful if someone is hurt, upset or feeling ill

10. Constantly fidgeting or squirming

11. Has at least one good friend

12. **Often fights with other children or bullies them**

13. Often unhappy, depressed or tearful

14. Generally liked by other children

15. Easily distracted, concentration wanders

16. Nervous or clingy in new situations, easily loses confidence

17. Kind to younger children

18. **Often lies or cheats**

19. Picked on or bullied by other children

20. Often offers to help others (parents, teachers, other children)

21. Thinks things out before acting

22. **Steals from home, school or elsewhere**

23. Gets along better with adults than with other children

24. Many fears, easily scared

25. Good attention span, sees work through to the end

Additional analyses

We used logistic regression models with clustered standard errors at the level of the individual and the pair (Stata v.13.1, logit2 program from http://www.kellogg.northwestern.edu/faculty/petersen/htm/papers/se/se_programming.htm). Our final model is shown in Table S2. No interactions were significant.

Table S2. Logistic regression output for primary analysis. Coefficients and Standard Errors for predictors of cooperation using two-dimensional clustering at the level of the individual and the pair.

	Coeff (SE)
Intercept	-0.239 (.20)
Repeated (1) or One Shot (0)	0.749 (.22)**
Instance	-0.038 (.01)***
Male (1)	-0.913 (.26)***
Conduct Problems Score	-0.165 (.08)*
Log Likelihood	-944.46
# Obs	1634
# Groups	539
# Clusters	58

*p < .05; ** p < .01; *** p < .001

Gender and Conduct Problems

To facilitate interpretation of the coefficients in the final model we calculated the odds ratios for the main predictors. Cooperation was lower for males so we used the reciprocal odds ratio (1/exp(coeff)). This showed that girls were 2.49 times more likely to cooperate than boys. Higher conduct problem scores also predicted lower cooperation, but Conduct is a continuous variable so we used 1-exp(coeff) to calculate the marginal decrease. This showed that a one point increase in conduct problems score predicted a 15% decrease in the likelihood of cooperation.

Cooperation over rounds

Cooperation declined over the testing sessions in both the one-shot and the repeated conditions, but overall cooperation remained higher in the repeated games (Figure S1.)

Figure S1. Cooperation over 30 rounds of play in the one-shot and repeated games.

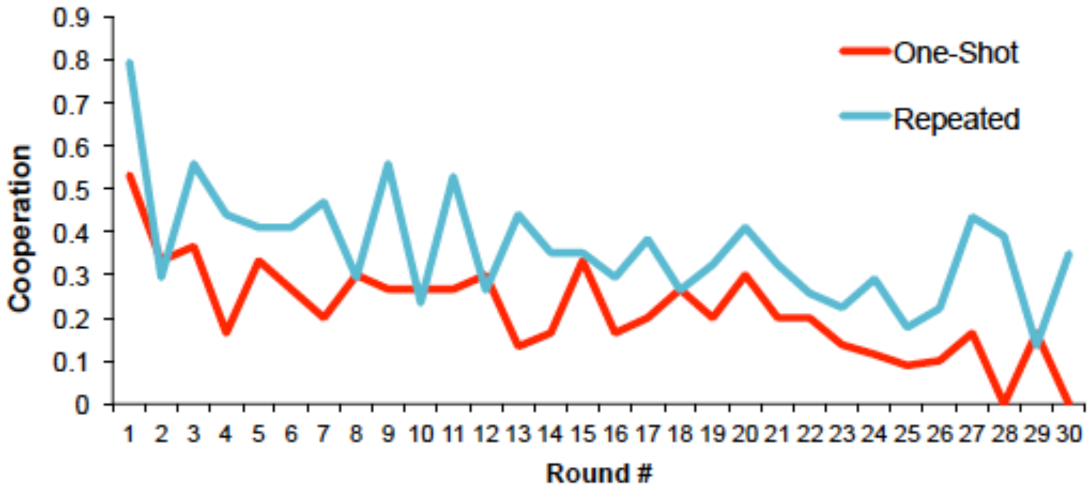

Supplement: Supporting Information [file srep14559-s1.pdf]
